# *Fasciola hepatica* Gastrodermal Cells Selectively Release Extracellular Vesicles via a Novel Atypical Secretory Mechanism

**DOI:** 10.3390/ijms23105525

**Published:** 2022-05-15

**Authors:** Adam P. S. Bennett, Eduardo de la Torre-Escudero, Susan S. E. Dermott, Lawrence T. Threadgold, Robert E. B. Hanna, Mark W. Robinson

**Affiliations:** 1 School of Biological Sciences, The Queen’s University of Belfast, Belfast BT9 5DL, UK; abennett12@qub.ac.uk (A.P.S.B.); e.delatorreescudero@qub.ac.uk (E.d.l.T.-E.); 2 Veterinary Sciences Division, Agri-Food and Biosciences Institute (AFBI), Stormont, Belfast BT4 3SD, UK; bob.hanna@afbini.gov.uk

**Keywords:** *Fasciola hepatica*, trematode, helminth, extracellular vesicle, secretion, proteome

## Abstract

The liver fluke, *Fasciola hepatica*, is an obligate blood-feeder, and the gastrodermal cells of the parasite form the interface with the host’s blood. Despite their importance in the host–parasite interaction, in-depth proteomic analysis of the gastrodermal cells is lacking. Here, we used laser microdissection of *F. hepatica* tissue sections to generate unique and biologically exclusive tissue fractions of the gastrodermal cells and tegument for analysis by mass spectrometry. A total of 226 gastrodermal cell proteins were identified, with proteases that degrade haemoglobin being the most abundant. Other detected proteins included those such as proton pumps and anticoagulants which maintain a microenvironment that facilitates digestion. By comparing the gastrodermal cell proteome and the 102 proteins identified in the laser microdissected tegument with previously published tegument proteomic datasets, we showed that one-quarter of proteins (removed by freeze–thaw extraction) or one-third of proteins (removed by detergent extraction) previously identified as tegumental were instead derived from the gastrodermal cells. Comparative analysis of the laser microdissected gastrodermal cells, tegument, and *F. hepatica* secretome revealed that the gastrodermal cells are the principal source of secreted proteins, as well as showed that both the gastrodermal cells and the tegument are likely to release subpopulations of extracellular vesicles (EVs). Microscopical examination of the gut caeca from flukes fixed immediately after their removal from the host bile ducts showed that selected gastrodermal cells underwent a progressive thinning of the apical plasma membrane which ruptured to release secretory vesicles en masse into the gut lumen. Our findings suggest that gut-derived EVs are released via a novel atypical secretory route and highlight the importance of the gastrodermal cells in nutrient acquisition and possible immunomodulation by the parasite.

## 1. Introduction

The liver fluke, *Fasciola hepatica*, is the causative agent of fasciolosis (liver fluke disease). Whilst historically regarded as a disease of livestock such as sheep and cattle, outbreaks in humans also make this disease a global health concern [1]. Mammalian hosts become infected by *F. hepatica* following ingestion of metacercariae which encyst on freshwater vegetation. Following excystment in the duodenum, the newly excysted juvenile flukes traverse the gut wall and enter the peritoneal cavity. After locating the liver, they migrate through the parenchymal tissue before entering the bile ducts where they mature into adults [2]. During the early stages of infection and migration, the parasite feeds principally on tissue [3]. However, adult flukes are obligate blood-feeders and use the amino acids derived from haemoglobin digestion to synthesise eggshell precursor proteins to support the production of up to 24,000 eggs per day [4].

The interface with the host’s blood is the fluke gut which is bifurcated and branches into blind-ending caeca that fill with blood, during feeding, due to the pumping action of the muscular pharynx [5]. The caeca are lined with gastrodermal cells which cycle between phases of secretion and absorption, thus exhibiting considerable variation in their cellular architecture [6,7]. Digestion by *F. hepatica* occurs predominately extracellularly in the gut lumen and, in their secretory phase, gastrodermal cells release large amounts of digestive enzymes, primarily cathepsins L1, L2, and L5, through exocytosis of zymogen-containing secretory granules [8,9,10,11,12]. Once exposed to the low pH within the gut lumen, the inactive cathepsin L zymogens undergo autocatalytic activation to release the mature (functional) enzyme domain [10,13]. The acidic conditions also relax the tertiary structure of haemoglobin, making it susceptible to cleavage into small peptides that can be absorbed by the gastrodermal cells for further hydrolysis by intracellular di- and amino-peptidases [10,14]. The by-products of this digestive process are periodically regurgitated into the host to prevent the accumulation of toxic haem [15].

We have recently shown that the gut and, to a lesser extent, the tegument and excretory system are the major sites of biogenesis and release of extracellular vesicles (EVs) in adult *F. hepatica* [16]. Intriguingly, an EV subtype (termed 15K EVs), derived from the gastrodermal cells, was found to contain the unprocessed 37 kDa cathepsin L zymogen suggesting that the vesicles are released intact into the gut lumen, a finding which was confirmed by transmission electron microscopy [17]. This indicates that cathepsin-containing secretory granules/vesicles do not exit the gastrodermal cell layer uniformly, and that some fuse with the apical plasma membrane to void their contents as soluble proteins in the gut lumen whilst others are released intact (as EVs) via an unknown secretory mechanism.

Here, we used laser microdissection (LMD) to isolate the gastrodermal cells and tegumental syncytium of adult *F. hepatica* for proteomic analysis, and we determined the relative contributions of these tissues to *F. hepatica* excretory/secretory products (ESP) by performing comparative analysis with the soluble and vesicular components of the *F. hepatica* secretome. Further histological and ultrastructural examination of adult *F. hepatica* showed that intact secretory vesicles are released from selected gastrodermal cells following rupture of the apical plasma membrane. These findings further our understanding of secretory processes in the fluke gastrodermis and provide a novel mechanism via which gut-derived EVs may be shed into host tissues.

## 2. Results

### 2.1. The Gastrodermal Cell Proteome Reflects Its Role in Secretion and Haematophagy

**Figure 1:**
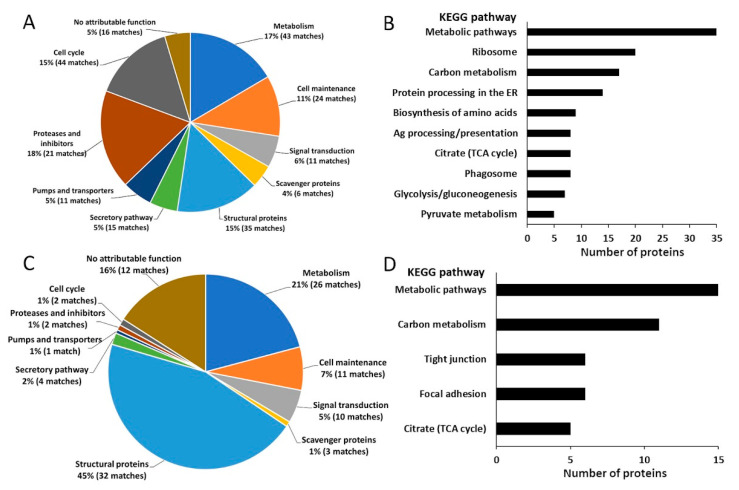
Different functional groups of proteins identified in the laser microdissected gut and tegument. (**A**) The percentage distribution of *F. hepatica* gastrodermal cell proteins within different functional groups based on their relative abundance. The number of matches corresponds to the number of different proteins that were detected in each category. (**B**) The number of proteins in gastrodermal cells associated with KEGG pathways. (**C**) The percentage distribution of *F. hepatica* laser microdissected tegument proteins within different functional groups based on their relative abundance. (**D**) The number of proteins in the LMD tegument associated with KEGG pathways.

As the gut and tegument represent key host–parasite interfaces and are the major sites of EV release by adult *F. hepatica* [16,18,19,20], these tissues were isolated by LMD and analysed by mass spectrometry (Figure S1). A total of 226 gastrodermal cell proteins were identified (>2 matched peptides in at least two out of four replicates) (Table 1 and Table S1A). Of these, 210 proteins had functional annotations, and 180 proteins could be assigned human orthologues, which were mapped to KEGG pathways (Figure 1). Whilst 63 of these proteins were also detected in the LMD tegument, 163 were exclusive to the gut (Table S1A). Notably, 69 of the proteins found in the gastrodermal cells were previously described in the surface protein fraction (SPF) generated by Haçariz et al. [21], but were not present in the LMD tegument (Table S1A). Likewise, 58 of the proteins described in the tegument extract of Wilson et al. [22] were not detected in the LMD tegument, but were present in the gastrodermal cells (Table S1A).

The proteins detected in the gastrodermal cells were grouped according to function (Table 1 and Table S1A), and Figure 1A shows the relative contributions of the functional groups to the total gastrodermal cell proteome. Proteases and protease inhibitors represented the most abundant group, comprising 18% (21 matches) of the total protein content. Amongst these were cathepsins L1, L2, and L5 which are secreted into the gut lumen and act in the extracellular digestion of haemoglobin [8,10,14,23], as well as seven aminopeptidases, including leucine aminopeptidase and aspartyl aminopeptidase, which complete the degradation of host-derived peptides [15]. Several of these proteolytic enzymes were described in previous proteomic analyses of the tegument [21,22], but were not detected in the LMD tegument proteome described here (Table 1). Protease inhibitors, including serpin, cystatin, and two kunitz-type proteinase inhibitors, were also detected in the gastrodermal cell proteome (Table 1), although these represented only 2% (four matches) of the total identified proteins. Other proteins associated with digestion and nutrient uptake were identified in the gastrodermal cells. Scavenger proteins constituted 4% of gastrodermal cell proteins (Figure 1A). These included proteins involved in the uptake of lipids (fatty acid-binding and lipoprotein-binding proteins) and the iron-binding protein ferritin (Table S1A). Moreover, 11 matches corresponded to membrane pumps and transporters, such as phosphatidylcholine transfer protein, vacuolar ATPase, and a Na^+^/K^+^ ATPase (Table 1).

Metabolic proteins were another abundant functional group (Figure 1A), and 55% of gastrodermal cell proteins mapped to KEGG metabolic pathways (Figure 1B). Proteins which function in both aerobic and anaerobic carbohydrate metabolic pathways were identified, with proteins from the malate dismutation pathway having a greater representation (8%, 18 matches) than TCA cycle-related proteins (5%, nine matches). Proteins of the protein biosynthesis machinery and secretion pathways were well represented in the gastrodermal cells. Transcriptional regulation (10 matches), protein synthesis (29 matches), and post-translational modification of proteins (five matches) constituted 15% of gastrodermal cell proteins (Figure 1A). Components of the ribosome alone represented 11% of the gastrodermal cell proteome, and ribosome (hsa03010) was the second most represented KEGG pathway (Figure 1B). In addition, 15 gastrodermal cell proteins were matched to the ER/Golgi-mediated secretory pathway (Table 1), and 14 proteins were mapped to the protein processing in the endoplasmic reticulum (hsa04141) KEGG pathway (Figure 1B). Proteins involved in atypical protein secretion were also identified in the gastrodermal cells (Table 1), including Rab11B and the neutral sphingomyelinase SMPD2, which are implicated in the biogenesis of exosomes (EVs derived from multivesicular endosomes), as well as ADP-ribosylation factor (ARF)1 and Ras-like GTP-binding protein Rho1 which promote the formation of microvesicles (EVs derived from the plasma membrane) [25].

Structural proteins comprised 15% (35 matches) of the gastrodermal cell proteome (Figure 1A) and included talin, abnormal long morphology protein 1, and extracellular matrix proteins which have not been described in either the tegument or the secretome of *F. hepatica* (Table 1). Signal transduction proteins, such as CaBP4 and 22.4 kDa tegument protein (Table 1), represented 6% (11 matches) of the total protein content, whilst cell maintenance proteins constituted 11% (24 matches) of the gastrodermal cell proteins (Figure 1A). Five percent (16 matches) of gastrodermal cell proteins were uncharacterised (Table S1A, Figure 1A), and the three most abundant were analysed using the I-TASSER homology modelling server [26]. These were predicted to resemble a vinculin-like protein (BN1106_s378B000167), which is involved in linkage of integrin to the cytoskeleton [27], a desmoplakin-like protein (BN1106_s2053B000154), which associates with junctional complexes [28], and α-2-macroglobulin (BN1106_s1647B000242), a protease inhibitor which can inhibit blood coagulation [29]. Eleven host proteins were identified, including cytoskeletal, nuclear, or heat-shock proteins (Table S1C).

### 2.2. The Proteome of the Tegument Supports Its Barrier Function

The protein composition of the LMD tegument differed markedly from the gastrodermal cells. A total of 102 proteins were identified in the LMD tegument, 39 of which were not detected in the gastrodermal cells (Table 2 and Table S1B). Ninety of the LMD tegument proteins had functional annotations (Figure 1C), 22 of which were not described in the previous *F. hepatica* tegument proteome dataset [22] or in the surface protein fraction (SPF) generated by Haçariz et al. [21] (Table 2 and Table S1B). Furthermore, 80 LMD tegument proteins could be assigned human orthologues and were mapped to KEGG pathways (Figure 1D). Structural proteins, including motor (10 matches), cytoskeletal (five matches), cytoskeletal-binding/modifying (eight matches), and extracellular matrix/cell adhesion proteins (nine matches), comprised the most abundant group (45%) of LMD tegument proteins (Figure 1C). Additionally, 18% of proteins mapped to focal adhesion (hsa04510) and tight junction (hsa04530) KEGG pathways (Figure 1D), reflecting the barrier function of the tegument.

Metabolic proteins comprised the second largest functional group (Figure 1C) and made up the highest proportion (72%) of proteins mapped to KEGG pathways (Figure 1D). As in the gastrodermal cells, proteins which function in both the malate dismutation pathway and the TCA cycle were identified (Table S1B) and comprised 8% (12 matches) and 11% (seven matches) of the total proteins, respectively. Proteins that could assist in the uptake of metabolic substrates from the host were also identified, such as two cholesterol-binding low-density lipoprotein receptors (Table S1B) and myoglobin, an oxygen carrier [30]. Other well-represented groups were cell maintenance proteins (including chaperones and antioxidants) and signal transduction proteins which constituted 7% (11 matches) and 5% (10 matches) of the total identified proteins, respectively (Figure 1C), with the calcium-binding proteins CaBP4 and calmodulin-like protein 2 being particularly abundant. Four vesicle-trafficking proteins were also identified, consistent with vesicle transport within the tegumental syncytium (Table S1B).

Uncharacterised proteins represented a considerable proportion (16%, 12 matches) of the LMD tegument proteome (Figure 1C). The three most abundant were analysed using I-TASSER [26]. As in the gastrodermal cells, the two most abundant were predicted to be desmoplakin (BN1106_s2053B000154) and vinculin-like proteins (BN1106_s378B000167), whilst the third was an orthologue of a bacterial ABC transporter ([31] BN1106_s487B000135) and also present in the gastrodermal cells (Table S1B). Additionally, six host proteins were detected in the LMD tegument (Table S1C).

### 2.3. Comparisons of the Tegument, Gut, and Secretome Indicate That the Gastrodermal Cells Are the Principal Source of Secreted Proteins

The *F. hepatica* secretome comprises soluble ESP and EVs which can be further separated into crude 15K EV and 120K EV populations by differential ultracentrifugation of parasite culture supernatants [17,24]. A total of 1105 proteins were identified in 15K EVs by combining the mass spectrometry results from this study (Table S2) and de la Torre Escudero et al. [24], whilst 256 proteins were described in 120K EVs [17,24] and 71 proteins in the soluble ESP [17]. To further explore the relative contributions of the gastrodermal cells and tegument to the *F. hepatica* secretome, a comparative analysis was performed between the protein profiles of these tissues and the secretome components described above. Figure S2A shows that the gastrodermal cells share 64% of their proteins with 15K EVs, 26% with 120K EVs, and 14% with the soluble ESP. Of the shared gastrodermal cell proteins, 68% (99 proteins) were exclusive (i.e., not also shared with the LMD tegument) to the gastrodermal cells and 15K EVs, 64% (37 proteins) were exclusive to the gastrodermal cells and 120K EVs, and 55% (17 proteins) were exclusive to the gastrodermal cells and soluble ESP, indicating that the gastrodermal cells are the source of these secretome components. Notably, all 18 proteases detected in the gastrodermal cells were previously described in the *F. hepatica* secretome: 11 in *F. hepatica* vomitus, 16 in 15K EVs, 13 in 120K EVs, and six in the soluble ESP (Table 1). Many gastrodermal cell proteins were shared across the various secretome components (Figure S2A), and six gastrodermal cell proteins (lysosomal Pro-X carboxypeptidase, glutathione *S*-transferase sigma class, cystatin-1, fructose-bisphosphate aldolase, cathepsin B4/5/7, and the acid-like sphingomyelinase, sphingomyelin phosphodiesterase-like 3; SMPDL3) were identified in all secretome components.

In comparison, the LMD tegument shared 77% of its proteins with 15K EVs, 36% with 120K EVs, and 18% with the soluble ESP (Figure S1B). Of those shared LMD tegument proteins, 42% (33 proteins) were exclusive (i.e., not also shared with gastrodermal cells) to the LMD tegument and 15K EVs, 43% (16 proteins) were exclusive to the LMD tegument and 120K EVs, and 22% (four proteins) were exclusive to the LMD tegument and soluble ESP, indicating that the tegument may be the source of these secretome components. Again, several LMD tegument proteins were shared between the EVs and non-vesicular ESP (Figure S2B), and four tegument proteins were detected in all secretome fractions: glutathione *S*-transferase sigma class, cystatin-1, fructose-bisphosphate aldolase, and an uncharacterised protein (BN1106_s6821B000024) which, when examined using I-TASSER, resembled cubilin, a co-transporter that facilitates the uptake of nutrients [32,33]. Glutathione *S*-transferase sigma class, cystatin-1, and fructose-bisphosphate aldolase were also found in the gastrodermal cells, signifying that these three proteins are common to the tegument, gastrodermal cells, and all ESP components (Table S1).

### 2.4. Microscopical Examination Suggests an Atypical Mechanism of Extracellular Vesicle Release by Some Gastrodermal Cells

**Figure 2:**
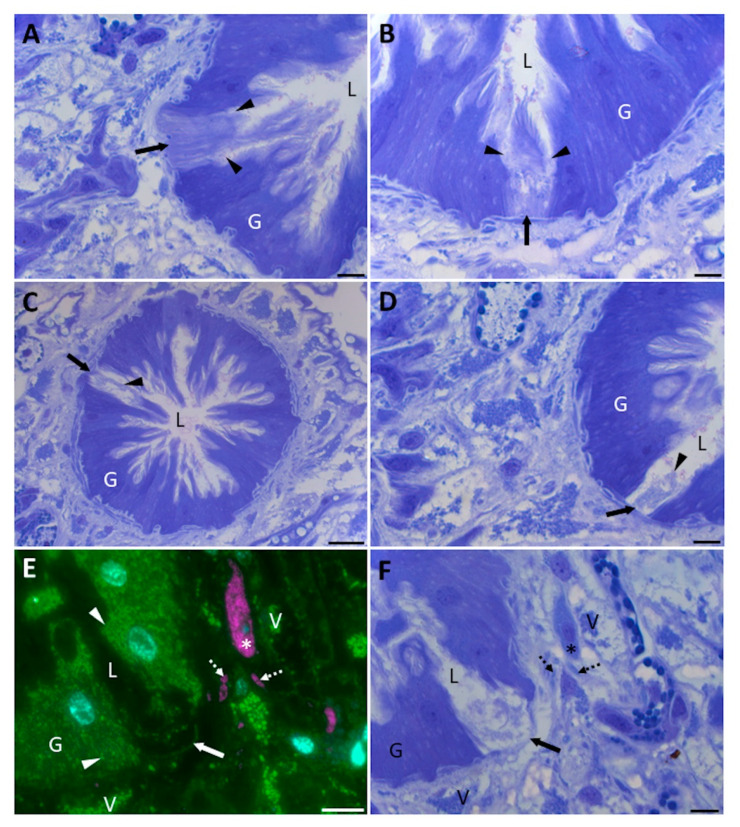
Individual gastrodermal cells of adult *F. hepatica* undergo progressive disintegration. (**A**–**D**) Toluidine blue staining showing gastrodermal cells at different stages of disintegration. Arrows point to the rupturing cells, and arrowheads indicate the disintegrating apical cell membrane (**A**,**B**) and residual material (**C**,**D**). (**E**) Immunostaining against FhRal-A (green) and tyrosinated α-tubulin (magenta) in the region proximal to a ruptured gastrodermal cell. Cell nuclei were counterstained with DAPI (cyan). Tyrosinated α-tubulin localises to vesicular structures in the parenchyma (dashed arrows) and packed within a specific cell (asterisk). FhRal-A localises to discrete puncta within gastrodermal cells which are concentrated at the cell apices (arrowheads), as well as groupings of vesicular structures (V) dispersedly packed within the sub-gastrodermal parenchyma. FhRal-A does not localise to the same cell labelled by the anti-tyrosinated α-tubulin antibody (asterisk). (**F**) Toluidine blue staining of the section shown in E shows that the structures positive for FhRal-A (V) stain dark blue (displaying basophilia), whilst those positive for tyrosinated α-tubulin (dashed arrows, asterisk) stain a lighter blue (displaying less basophilia). G, gut; L, lumen. Scale bars: 7.5 µm (**A**,**B**,**D**,**E**), 25 µm (**C**,**F**).

The gastrodermal cells of adult *F. hepatica* secrete cathepsin L as a soluble 37 kDa zymogen that rapidly undergoes autocatalytic activation within the gut lumen following its release by classical exocytosis of secretory vesicles (i.e., fusion of the vesicles with the apical plasma membrane and discharge of soluble contents into the gut lumen [9,12]). However, these cells also release intact 15K EVs containing unprocessed cathepsin L zymogens which can be similarly activated upon lysis of the vesicles in vitro [17]. Whilst proteins involved in the classical ER/Golgi secretory pathway for the release of soluble proteins were well represented in the gastrodermal cell proteome (Figure 1A, Table 1), little was revealed about how these cells could release intact 15K EVs. To examine how the gastrodermal cells might release intact vesicles, adult flukes were fixed immediately after their removal from the bile ducts (to preserve their in situ morphology) and then processed for histological observation. This revealed that some individual gastrodermal cells were less basophilic than neighbouring cells within the gut caeca and appeared to be in the process of breaking down (Figure 2A–D).

Immunolabelling of the gastrodermal region surrounding these cells showed different subtypes of vesicles positive for FhRal-A and tyrosinated α-tubulin in close proximity to the gut caeca (Figure 2E). The FhRal-A antibody labelled vesicles within gastrodermal cells and dispersed within sub-gastrodermal parenchymal cells (Figure 2E), as described previously [16,24]. Conversely, the anti-tyrosinated α-tubulin antibody labelled compact discoid structures and vesicles tightly packed within a discrete cell type in the parenchyma which did not co-localise with FhRal-A (Figure 2E). Toluidine blue staining of these immunostained structures suggested that they may be different vesicle subtypes (Figure 2F). The intracellular vesicular structures labelled by FhRal-A appeared more basophilic (dark blue) and were located within parenchymal cells with a pale (and, hence, weakly basophilic) cytoplasm, typical of PC2 type parenchymal cells [16,34]. Conversely, tyrosinated α-tubulin-positive structures stained light blue and filled the entire cytoplasm of their resident cell (Figure 2F).

**Figure 3:**
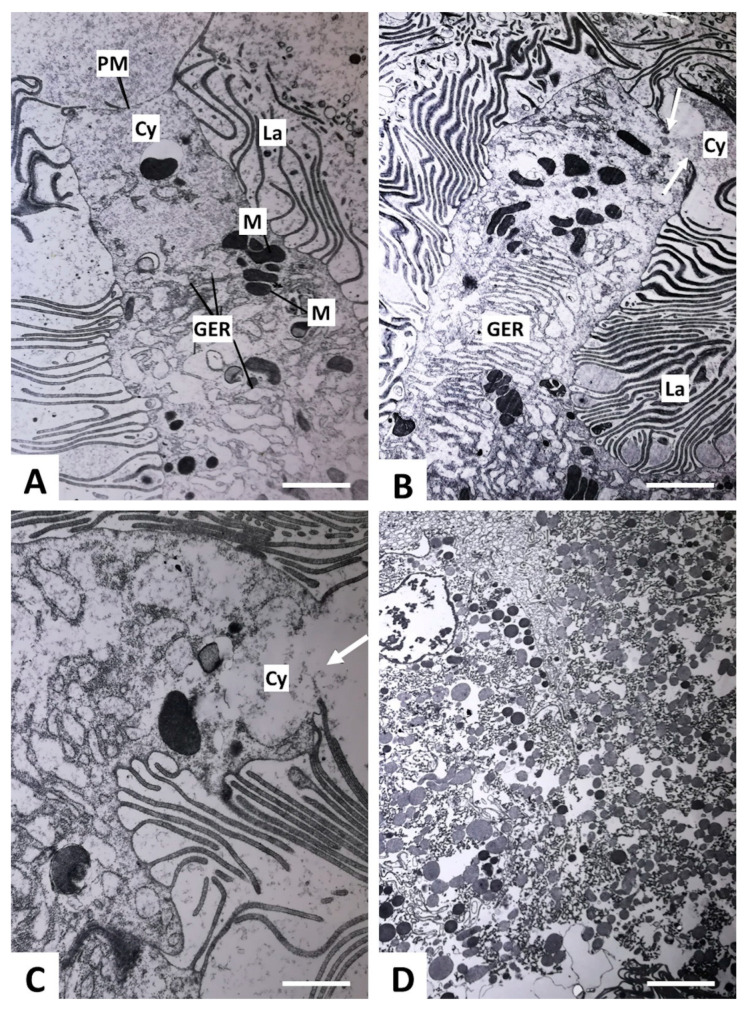
Transmission electron micrographs showing progressive breakdown of the gastrodermal cells of adult *F. hepatica*. (**A**) The apex of a columnar gastrodermal cell. The lamellae (La) are spaced far apart, and the apical plasma membrane (PM) appears very thin. (**B**) The apical plasma membrane is broken (arrows) and the cell has begun to leak apical cytoplasm (Cy) into the gut lumen. (**C**) High-power image of a break (arrow) in the apical plasma membrane. (**D**) An array of membrane fragments, mitochondria, and vesicles are released into the gut lumen. GER, granular endoplasmic reticulum; M, mitochondria. Scale bars: 5 µm (**A**,**B**), 2.5 µm (**C**), and 10 µm (**D**).

Transmission electron microscopy of the gastrodermal cells showed that some cells displayed progressive thinning of the apical plasma membrane coincident with reduced numbers of lamellae (Figure 3A). The apical membrane was seen to rupture in some cells (Figure 3B,C), which resulted in the leakage of cell contents (largely cytoplasm and secretory vesicles which typically accumulate within the apical region of the cell) into the gut lumen (Figure 3D).

## 3. Discussion

The gastrodermal cells and tegument represent the main cellular interfaces between *F. hepatica* and the host [15], and they are also sites of EV biogenesis and release from the adult flukes [16,19]. Therefore, LMD was used to generate the first biologically exclusive tissue fractions of the tegument and gastrodermal cells from adult flukes, for proteomic analysis, in order to shed light on the molecular processes that occur at these sites.

Of the 265 proteins identified in this study, 163 proteins were exclusive to the LMD gastrodermal cells and 39 proteins were exclusive to the LMD tegument, showing that each of these interfaces has a distinct proteome. It was also found that the protein composition of the LMD tegument differed from the adult *F. hepatica* tegumental proteomes described previously [21,22]. Such variation is likely due to differing techniques of tegument enrichment and detergent extraction of proteins. The advantages of LMD over previous isolation methods, however, are notable. Fixing flukes immediately after their removal from the bile ducts preserves temporal and spatial levels of protein expression, akin to those observed in vivo [35]. Furthermore, by generating biologically exclusive tissue samples, we show that up to 25% [22] and 33% [21] of proteins previously identified as tegumental (e.g., proteases, nuclear proteins and ribosomal proteins) may actually be contaminants from the gastrodermal cells which would also be removed during traditional freeze–thaw and detergent extraction steps [21,22]. This study, therefore, validates the use of LMD for helminth sub-proteome analysis.

Consistent with their digestive function, proteases were the most abundant functional group in the gastrodermal cells (representing 16% of the total protein content), and substantial amounts of these proteases are secreted by adult *F. hepatica* (estimated at 5–10 µg/h of cathepsin L cysteine proteases per fluke [15]). Accordingly, the machinery involved in the synthesis, processing, and secretion of these proteases through the classical secretory pathway was well represented in the gastrodermal cell proteome, emphasising that these are highly secretory cells. All the identified proteases were previously found in the parasite secretome and participate in extracellular digestion of host haemoglobin in the gut lumen [10]. However, while cathepsins L dominate the secretome [14,23,36], leucine aminopeptidase was the most abundant protein in the gastrodermal cells. Immunogold labelling has shown that leucine aminopeptidase localises to the gastrodermal cell lamellae, indicating that it functions intracellularly to complete digestion of absorbed haemoglobin-derived peptides in the cytosol [15,37]. The numerous proton pumps identified in the gastrodermal cells could facilitate digestion by maintaining an acidic pH in the gut lumen which serves to activate cathepsin Ls and relax the structure of haemoglobin, making it susceptible to proteolysis [10].

Other gastrodermal cell proteins may also be indirectly involved in haematophagy. The protease natterin-4 is an orthologue of a fish venom protein that prevents human blood coagulation [38]. Similarly, the abundant uncharacterised protein BN1106_s1647B000242 was a predicted structural orthologue of α-2-macroglobulin, another anticoagulant [29]. Natterin-4 has also been detected in *F. hepatica* EVs [17,24], and BN1106_s1647B000242 has a predicted N-terminal signal peptide, indicating that these proteins are secreted into the gut lumen and may promote blood feeding by preventing coagulation in the fluke gut. Interestingly, both of these proteins are targets of an immune response in resistant mammalian hosts [39,40] and are fluke-specific, suggesting that they may be useful vaccine candidates.

The tegument of *F. hepatica* protects internal fluke tissue from the harsh environment of the bile ducts [15]. The predominance of cytoskeletal proteins in the LMD tegument supports this barrier function, whilst the cytoskeletal-modifying enzymes and cell maintenance proteins could function in repairing damaged tissue and proteins to maintain tegument integrity. Similarly, cystatin-1 and serpin (cysteine and serine protease inhibitors, respectively) could protect the tegument against host proteases, but may additionally regulate endogenous proteases such as cathepsins which have been identified in *Fasciola*-infected bile [36]. The abundance of metabolic proteins identified in the LMD tegument reflects the large number of mitochondria within the syncytium [41]. Whilst anaerobic carbohydrate metabolism (namely malate dismutation) is believed to predominate in adult *F. hepatica* [42,43], previous studies focused on whole flukes without taking into account the metabolic capabilities of specific tissues [44]. Using LMD, this study identified components of the aerobic TCA cycle, indicating that aerobic metabolism may also occur in the tegument (and gastrodermal cells). This supports the hypothesis that sufficient oxygen reaches the outer surface of adult *F. hepatica* such that aerobic metabolism can proceed [42,44]. The oxygen carrier myoglobin (detected in the LMD tegument) may enhance oxygen delivery to the tissue, although this requires further investigation.

The current proteome comparisons and previous experimental data suggest that both the tegument and the gastrodermal cells secrete EVs that comprise subsets of the crude 15K EV and 120K EV populations that are enriched following ultracentrifugation of fluke culture supernatants [16]. Although proteins involved in the biogenesis of exosomes (EVs derived from multivesicular endosomes) are conserved in *F. hepatica* [45] and structures resembling multivesicular endosomes have been observed in the tegumental syncytium [20], no exosome biogenesis proteins were identified in the LMD tegument. However, motor proteins and other vesicle trafficking proteins were detected and shared with 120K EVs [17,24]. These could be involved in the transport of multivesicular endosomes along microtubules to the APM for 120K EV release or in the transfer of T1/T2 secretory bodies to the glycocalyx. Exosome biogenesis proteins may instead be expressed in tegumental cell bodies or in sub-tegumental parenchymal cells [16], which were not isolated here by LMD. Although technically challenging, fluorescent antibodies targeting markers of tegumental cell bodies, such as FhTeg1 [16], could be used in combination with LMD [46,47] to specifically isolate tegumental cell bodies for transcriptomic analysis to reveal more about their secretory processes.

Proteins exclusively shared between the tegument and 15K EVs included cytoskeletal remodelling proteins and calcium-sensitive signal transduction proteins, which are associated with the generation of microvesicles at the plasma membrane of cells. This supports the proposition that an increase in intracellular Ca^2+^ concentration could activate a signalling cascade that induces cytoskeletal reorganisation and enables budding of microvesicles from the tegument apical plasma membrane [20]. The release of microvesicles (previously described as ‘blebs’) from the tegument is thought to contribute to parasite survival in response to external stresses and could even be involved in the sequestration and removal of anthelmintics from the parasite [20,48]. Further characterisation of these processes may explain how flukes repair the damaged tegument and could perhaps be exploited to increase the efficacy of existing anthelmintics.

Numerous proteins were shared exclusively between the gastrodermal cells and *F. hepatica* EVs, which supports our previous findings that the gut is the principal source of these vesicles [16]. Amongst the shared proteins, those associated with both exosome (sphingomyelinases and Rab11) and microvesicle (ARF1, Rho1, and calpain) formation were identified. However, as for the LMD tegument, many members of the EV biogenesis pathways found in *F. hepatica* EVs were not present in the gastrodermal cells. These proteins may not have been detected because of their low expression levels in *F. hepatica* [17] such that they were not suitably extracted from the fixed tissue sections. Previous in vitro studies showed that the release of 120K EVs from adult *F. hepatica* was sensitive to GW4869, a chemical inhibitor of sphingomyelin-driven exosome formation [16], suggesting that these EVs are “exosome-like” and may derive from the endosomal system. In contrast, the release of gut-derived 15K EVs was largely unaffected by exposure to GW4869 in vitro [16], which suggests they may form via canonical vesicle biogenesis pathways. Indeed, proteins shared between the 15K EVs and the LMD gastrodermal cells include coatomers and Sec23 which are more commonly associated with intracellular vesicles formed via the ER/Golgi secretory pathway [49]. This supports the proposal that some 15K EVs may be released intact, via an atypical form of secretion, whilst others fuse with the apical plasma membrane and release their contents into the gut lumen via exocytosis.

Histological examination of the adult fluke gastrodermis showed that some individual cells lining the gut caeca (consistently observed across multiple tissue sections and from several individual flukes) displayed reduced cellular integrity or were absent altogether. At the ultrastructural level, these cells were seen to undergo a progressive thinning of their apical plasma membrane before rupturing to release intact secretory vesicles into the gut lumen. The reason behind such an atypical secretory mechanism remains unclear although it would enable delivery of gut-derived EVs en masse into the host bile/bloodstream during the regular regurgitation of fluke ESPs that occurs during feeding [22]. Given the variety of protein and RNA cargo associated with *F. hepatica* EVs [50,51,52], it is conceivable that EVs released in this way may exert immunomodulatory or other effects on host cells at locations quite distant from their bile duct niche. Whether the ruptured gastrodermal cells are degraded entirely and replaced with new cells is unclear. However, there are numerous examples of cells that are capable of repairing damaged plasma membrane (i.e., wound healing to prevent further loss of cytoplasm/cell volume) and then regenerating lost structures (reviewed by [53]); hence, this may also be feasible within the *F. hepatica* gastrodermis. It is noteworthy that two fusogenic proteins (EHD1 and myoferlin) are found on the outer membrane surface of the gut-derived *F. hepatica* 15K EVs [24]. Since an additional bio-membrane for repair of plasma membrane damage is often supplied by resident cytoplasmic vesicles [54], it possible that these proteins could mediate fusion and repair of the ruptured gastrodermal cell apical plasma membrane.

By creating unique and distinct proteome datasets for the gastrodermis and tegumental syncytium, this study unveiled the contribution of each of these tissues to secretory processes in the liver fluke and identified a novel route for export of gut-derived EVs into host tissues. Knowledge of vesicle formation, trafficking, and secretion is critical to understand how helminths interact with their mammalian hosts and repair damaged cells. Accordingly, our findings may guide new treatments for parasite infection to disrupt these key pathways.

## 4. Materials and Methods

### 4.1. Laser Microdissection

Paraffin-embedded adult *F. hepatica* (prepared as described by Hanna et al. [55]) were cut into thick (10 µm) sections (to maximise tissue recovery), placed on polyester membrane steel-framed slides (Leica Microsystems Ltd., Newcastle Upon Tyne, UK), and lightly stained in 1% (*w*/*v*) toluidine blue for 2 s at room temperature (18–21 °C) to allow visualisation of the tissue. For LMD, the gastrodermal cells and tegumental syncytium were excised from the tissue sections using an ultraviolet laser on either a LMD7 (Leica Microsystems Ltd., Newcastle Upon Tyne, UK) or PALM MicroBeam Laser Micro-dissector (Zeiss) system and collected in 0.5 mL microcentrifuge tubes. LMD consisted of four independent experiments.

### 4.2. Mass Spectrometry of Laser Microdissected Tissue

LMD samples were sent to the Fingerprints Proteomics Facility at the University of Dundee for mass spectrometry analysis. To extract proteins from the paraffin-embedded tissue, 90 µL of lysis buffer (4% SDS, 100 mM DTT in 100 mM Tris-HCl, pH 8.5) was added to the tissue, which was then heated for 1 h at 95 °C. Following this, the samples were processed using the filter-aided sample preparation (FASP) protocol, as described by Wiśniewski et al. [56]. Extracted proteins were double digested with trypsin (2 × 2.5 µg) and the resulting peptides were desalted and resuspended in 30 µL of 1% formic acid. Then, 15 µL of the resulting suspension was delivered to an analytical column (Eksigen C18-CL NanoLC Column, 3 µm; 75 µm × 15 cm), and the fractionated peptides were analysed in a nanoESI QqTOF mass spectrometer (5600 TripleTOF, ABSCIEX) as described by Cwiklinski et al. [17]. Peak list files were generated by Protein Pilot v4.5 (Applied Biosystems) using default parameters and exported to Mascot v2.4.1 (Matrix Science) for database searching.

### 4.3. Database Searching

Mascot was set up to search a database comprising the gene models identified within the *F. hepatica* genome (version 1.0, 101,780 entries [57]) assuming trypsin digestion with one missed cleavage permitted. The *F. hepatica* gene model sequences can be accessed through WormBase ParaSite (http://parasite.wormbase.org/ (accessed on 1 November 2017)) under accession PRJEB6687. Mascot was searched with a fragment ion mass tolerance of 0.60 Da and a parent ion tolerance of 10.0 ppm. Carbamidomethylation of cysteine was specified in Mascot as a fixed modification. Gln- > pyro-Glu of the N-terminus, deamidation of asparagine and glutamine, oxidation of methionine, dioxidation of methionine, and acetylation of the N-terminus were specified in Mascot as variable modifications. An additional search against the *Rattus norvegicus* gene models (http://www.ensembl.org/Rattus_norvegicus/Info/Index (accessed on 1 November 2017)) was run to identify potential host proteins.

### 4.4. Criteria for Protein Identification

Scaffold (version Scaffold_4.11.0, Proteome Software Inc., Portland, Oregon) was used to validate MS/MS-based peptide and protein identifications. Peptide identifications were accepted if they could be established at >95.0% probability by the Scaffold Local FDR algorithm. Protein identifications were accepted if they could be established at >99.0% probability and contained at least two identified peptides. Protein probabilities were assigned by the Protein Prophet algorithm [58]. Proteins that contained similar peptides and could not be differentiated on the basis of MS/MS analysis alone were grouped to satisfy the principles of parsimony. Proteins sharing significant peptide evidence were grouped into clusters, and only those with at least two unique peptide matches present in two or more of the four replicates were accepted.

### 4.5. Functional Annotation of Proteins

Proteins were manually grouped into functional classifications on the basis of their Uniprot annotations (www.uniprot.org (accessed on 1 November 2017) [59]). Proteins were also subjected to KEGG pathway analysis [60] using the DAVID functional annotation tools (https://david.ncifcrf.gov/home.jsp (accessed on 1 November 2017) [61,62]). Since these tools are currently not supported for *F. hepatica*, human orthologues (identified using BLASTp with an E value <1 × 10^−5^ as a cut-off) were used to perform the analysis. A minimum of five members was used as the acceptance threshold for a KEGG pathway.

### 4.6. Isolation and Mass Spectrometry Analysis of Adult F. hepatica 15K Extracellular Vesicles

Adult flukes were obtained from the bile ducts of sheep immediately after slaughter at a local abattoir (Dungannon, Northern Ireland). After thoroughly washing the flukes in PBS containing 0.1% (*w*/*v*) glucose at 37 °C, parasites that had voided their gut contents were maintained in groups of 50 at 1 worm/mL in RPMI-1640 culture medium containing 0.1% (*w*/*v*) glucose, 100 U/mL penicillin and 100 µg/mL streptomycin (Sigma-Aldrich, Gillingham, UK) for 5 h at 37 °C to collect secretions. After 5 h incubation, EVs were isolated from the culture media by differential centrifugation as described by de la Torre-Escudero et al. [24] to generate a post-15,000× *g* pellet (15K EVs). EV pellets were resuspended in filter-sterilised PBS, snap-frozen, and stored at −80 °C until further analysis. Three biological replicates (15 µg each sample) of the *F. hepatica* 15K EVs were run on reducing NuPage Novex 4–12% Bis-Tris gels (ThermoFisher). Gel lanes were cut into three sections, and proteins were reduced with 2 mM DTT in 50 mM NH_4_HCO_3_ at 60 °C for 20 min and alkylated with 5 mM iodoacetamide at room temperature in the dark for 30 min. Samples were in-gel digested with 100 ng/µL sequencing-grade trypsin (Promega) overnight at 37 °C. Peptides were solubilised in 2% formic acid and analysed by mass spectrometry as described above.

### 4.7. Histology and Immunofluorescent Labelling of F. hepatica Tissue Sections

Adult *F. hepatica* were collected from the bile ducts of sheep (as described above) and immediately placed in PBS containing 4% paraformaldehyde (Sigma-Aldrich) for 4 h at room temperature. Flukes were subsequently embedded in JB-4 resin (Sigma-Aldrich) according to the manufacturer’s instructions. Semi-thin (2 µm) sections were cut on a pyramitome and mounted on clean glass slides and stained with 1% (*w*/*v*) toluidine blue for 30 s or subjected to immunofluorescent labelling according to Bennett et al. [16]. Briefly, tissue sections were washed in PBS then incubated overnight in primary antibody in antibody diluent (AbD: PBS containing 0.2% (*v*/*v*) Triton X-100) at room temperature. The anti-tyrosinated α-tubulin mouse monoclonal (TUB-1A2; Sigma-Aldrich) was used at a 1:100 dilution whilst the rabbit polyclonal antibody (Genscript) raised against FhRal-A was used at 10 µg/mL. As a negative control, tissue sections were also incubated with pre-immune serum in AbD. The sections were then washed three times in AbD before incubation in an appropriate secondary antibody-FITC/-TRITC conjugate at a 1:200 dilution in AbD for 1 h at room temperature. The sections were counterstained in 300 nM DAPI (ThermoFisher) and mounted in glycerol containing 10% (*v*/*v*) PBS and 0.1 M propyl gallate (Sigma-Aldrich). Sections were viewed under a Leica DM2500 fluorescent microscope.

### 4.8. Processing of F. hepatica for Transmission Electron Microscopy

Flukes were fixed and processed for transmission electron microscopy as described by Gallagher and Threadgold [63]. Briefly, whole worms were fixed in 2% (*w*/*v*) glutaraldehyde in phosphate buffer (pH 7.4) for 2 h. Post fixation, the tissue was washed then placed in 1% osmium tetroxide for 2 h, washed again before infiltration, and embedding in Maraglas epoxy resin. Ultrathin sections (80 nm) were mounted on uncoated copper grids then stained with uranyl acetate and aqueous lead citrate. The grids were viewed using an A.E.I. EM6B TEM operating at 80 kV.

## Figures and Tables

**Table 1:** Proteases, protease inhibitors, pumps, transporters, and structural, signal transduction, and secretory pathway proteins identified in the gastrodermal cells of adult *F. hepatica* and compared with proteins identified in the laser microdissected (LMD) tegument (this study), as well as previously reported surface, tegument, and excretory/secretory proteins (ESP). A dot indicates a shared protein. R1–4 = replicates 1–4. ^1^ This study, ^2^ [21], ^3^ [22], ^4^ [17], ^5^ [24].

Protein	Identifier	Gastrodermal Cell				LMD Tegument ^1^	Previously Detected						
		Unique Peptides					Surface Protein Fraction ^2^	Tegument ^3^	Vomitus ^3^	ESP Minus Vomitus ^3^	Soluble ESP ^4^	15K EVs ^1,5^	120K EVs ^4,5^
		R1	R2	R3	R4								
**Proteases and inhibitors**													
*Proteases*													
Leucine aminopeptidase 2	BN1106_s617B000566	15	11	18	17		●	●	●			●	●
Xaa-Pro dipeptidase (M24 family)	BN1106_s468B000343	6	4	5	13		●		●	●		●	●
Leucine aminopeptidase 2	BN1106_s617B000567	5	4	5	6		●	●	●			●	●
Lysosomal Pro-X carboxypeptidase	BN1106_s1620B000120	5	4	5	3				●	●	●	●	●
Leucine aminopeptidase 1	BN1106_s7079B000034	5	4	4	12		●	●	●			●	●
Cathepsin B6/8	BN1106_s793B000177	5	3	4	5						●	●	●
Cathepsin L1	BN1106_s8490B000026	4	4	5	5		●	●	●	●	●	●	●
Cathepsin L2	BN1106_s8098B000020	3	3	5	7		●	●	●		●	●	●
Natterin-4 (DM9 domain-containing protein)	BN1106_s586B000374	3	3	5	6		●	●				●	●
Cathepsin L1	BN1106_s10332B000010	3	2	3	1		●	●	●	●			
Cathepsin L5	BN1106_s4636B000039	2	3	2	5				●	●			
Cathepsin B4/5/7	BN1106_s13444B000002	2	2	3	5						●	●	●
Cathepsin L5	BN1106_s6354B000017	2	2	2	2				●	●		●	●
Cysteine protease-related protein	BN1106_s1772B000188	1	2	2	5							●	●
Aspartyl aminopeptidase	BN1106_s2165B000367	1	2	2	4							●	
Legumain-like	BN1106_s1861B000097	1	1	3	4				●	●	●	●	●
Mitochondrial processing peptidase	BN1106_s37B000342	1	0	2	2							●	
*Protease inhibitors*													
Cystatin-1	BN1106_s4651B000094	4	3	4	7	●	●				●	●	●
Serpin	BN1106_s3864B000104	3	2	5	2	●	●			●		●	
FH-KTM kunitz-type proteinase inhibitor	BN1106_s318B000274	2	2	3	2			●	●	●	●		●
FH-KTM kunitz-type proteinase inhibitor	BN1106_s8826B000029	1	2	2	2			●	●	●			●
**Structural proteins**													
*Motor/cytoskeletal*													
Myosin heavy chain	BN1106_s323B000258	16	12	19	29	●							
Paramyosin	BN1106_s1922B000122	8	4	11	21	●							
Filamin	BN1106_s296B000186	5	0	5	6	●	●				●		
Actin	BN1106_s658B000223	4	4	6	9		●	●		●		●	
Filamin-C	BN1106_s1515B000336	4	3	3	5	●						●	
Myosin heavy chain	BN1106_s323B000257	3	1	4	5	●	●						
Myosin heavy chain	BN1106_s3182B000117	3	0	2	9	●	●					●	
Beta-tubulin 4	BN1106_s1153B000359	2	2	3	3		●	●				●	
Actin	BN1106_s101B000531	2	2	3	2		●	●		●			
Alpha-tubulin 2	BN1106_s925B000543	2	1	2	0		●	●					
Talin	BN1106_s149B000360	2	0	2	1								
Alpha-actinin sarcomeric	BN1106_s4069B000247	1	2	1	4	●	●	●				●	●
Tropomyosin	BN1106_s647B000405	1	1	5	3		●						
Lamin	BN1106_s1106B000091	1	1	2	5								
Tropomyosin-2	BN1106_s4130B000080	1	1	1	7	●	●					●	
Tubulin beta-3	BN1106_s4860B000047	1	1	0	7		●	●				●	
Titin	BN1106_s98B000745	1	0	2	2			●					
*Cytoskeletal-binding/modifying*													
Spectrin beta chain, brain 4	BN1106_s4255B000066	7	3	8	8	●	●						
Merlin/moesin/ezrin/radixin	BN1106_s1300B000145	6	8	9	18		●	●				●	●
Ezrin-radixin-moesin-binding phosphoprotein 50	BN1106_s1037B000175	4	3	6	6		●					●	
Calpain (C02 family)	BN1106_s204B000249	4	0	4	7	●		●				●	
Rho GDP-dissociation inhibitor	BN1106_s4672B000098	2	2	3	1		●						
Myophilin	BN1106_s3747B000111	2	1	2	3		●	●	●	●			
Ras-like GTP-binding protein Rho1	BN1106_s1908B000177	1	2	2	1			●				●	●
Calponin homolog	BN1106_s2140B000163	1	1	4	2	●	●						
Translationally controlled tumour protein	BN1106_s17035B000006	3	2	3	5		●					●	
Abnormal long morphology protein 1	BN1106_s1644B000226	0	0	2	2								
*Extracellular matrix/cell adhesion*													
Heparan sulphate proteoglycan	BN1106_s25B000189	5	3	12	10	●	●				●		
Alpha 1 type IIA collagen	BN1106_s849B000266	3	2	4	4	●							
Collagen alpha-1(V) chain	BN1106_s457B000392	2	2	2	3	●							
Fasciclin-1	BN1106_s100B000380	2	2	2	2								
Collagen alpha-1(V) chain	BN1106_s2714B000202	2	0	2	3	●							
Fasciclin I-like protein	BN1106_s1922B000120	1	1	3	3	●					●		
Collagen alpha-2(I) chain	BN1106_s104B000457	1	1	2	2	●							
Collagen alpha-1(V) chain	BN1106_s849B000265	1	0	2	3	●							
**Signal transduction**													
*Signalling*													
Tyrosine 3-monooxygenase	BN1106_s3172B000053	4	4	6	6	●	●	●				●	
14-3-3 epsilon	BN1106_s686B000273	4	2	4	5	●	●	●		●		●	
14-3-3 protein beta	BN1106_s3904B000042	2	2	1	3		●	●			●	●	●
Nucleoside diphosphate kinase	BN1106_s344B000191	1	1	2	2		●			●		●	
Protein CBR-FTT-2	BN1106_s4074B000042	1	1	2	2			●					
Camp-dependent protein kinase type II-alpha	BN1106_s417B000229	1	0	2	3							●	●
*Calcium-binding*													
CaBP4	BN1106_s214B000744	6	4	8	11	●	●	●		●		●	●
22.4 kDa tegument protein	BN1106_s214B000748	4	4	4	4		●	●				●	
Tegumental calcium-binding EF-hand protein	BN1106_s214B000742	1	1	2	3	●	●	●		●		●	
*Ubiquitination pathway*													
Ubiquitin-activating enzyme E1	BN1106_s5276B000036	1	3	1	6	●	●					●	
Ubiquitin-protein ligase BRE1	BN1106_s208B000185	1	1	2	3	●	●					●	●
**Secretory pathway**													
*ER/Golgi apparatus-related*													
Venom allergen-like 11 protein	BN1106_s1956B000118	5	4	5	6							●	
Calreticulin	BN1106_s2673B000071	2	1	3	6							●	
Transitional endoplasmic reticulum ATPase	BN1106_s5369B000082	2	1	1	2		●					●	●
Preprotein translocase secy subunit Sec61	BN1106_s2995B000137	1	1	2	2								
Atlastin-1	BN1106_s3190B000098	0	1	2	2								
*Vesicle biogenesis and trafficking*													
Annexin	BN1106_s819B000364	8	8	9	11	●	●	●		●	●	●	●
Annexin	BN1106_s945B000218	4	2	5	8		●	●		●	●	●	●
Coatomer subunit beta	BN1106_s771B000471	3	0	3	2							●	
ADP-ribosylation factor 1	BN1106_s4512B000085	2	2	2	2		●					●	
SAR1 gene homolog B	BN1106_s1405B000146	2	2	2	0								
Coatomer protein complex subunit alpha	BN1106_s649B000500	2	1	2	3							●	
Annexin	BN1106_s500B000161	1	3	2	5	●	●	●		●		●	●
Coatomer subunit gamma	BN1106_s5131B000049	1	0	3	7							●	
Ras-related protein Rab-11B	BN1106_s844B000259	1	0	3	3			●				●	●
Protein transport protein Sec23A	BN1106_s2174B000116	1	0	2	3							●	
**Pumps and transporters**													
Smdr2	BN1106_s274B000296	8	7	12	18						●	●	●
ATP synthase subunit beta	BN1106_s1866B000129	7	5	7	8		●	●				●	
ATP synthase subunit alpha	BN1106_s4332B000087	6	6	5	10		●	●		●		●	●
ATP:ADP antiporter	BN1106_s3313B000078	2	2	5	8	●	●					●	
V-type H+-transporting ATPase subunit A	BN1106_s2110B000156	2	2	3	4		●					●	
Innexin	BN1106_s503B000225	2	2	2	2								
Phosphatidylcholine transfer protein	BN1106_s538B000493	2	2	1	2							●	●
Sodium potassium transporting ATPase alpha subunit	BN1106_s521B000167	2	1	1	3		●					●	●
Vacuolar H+-ATPase SFD subunit	BN1106_s2350B000136	1	2	2	2			●				●	
Vacuolar ATP synthase subunit e	BN1106_s1399B000513	1	1	2	3			●				●	
ATP synthase subunit beta vacuolar	BN1106_s1633B000182	0	0	3	5			●				●	

**Table 2:** Structural and signal transduction proteins identified in the laser microdissected (LMD) tegument of adult *F. hepatica* and compared with proteins identified in the gastrodermal cells (this study), as well as previously reported surface, tegument, and excretory/secretory proteins (ESP). A dot indicates a shared protein. Asterisks indicate the proteins that were not described in the previous *F. hepatica* tegument proteome dataset [22] or in the surface protein fraction (SPF) generated by Haçariz et al. [21]. R1–4 = replicates 1–4. ^1^ This study, ^2^ [21], ^3^ [22], ^4^ [17], ^5^ [24].

Protein	Identifier	LMD Tegument				Previously Detected							
						Gastrodermal Cells ^1^	Surface Protein Fraction ^2^	Tegument ^3^	Vomitus ^3^	ESP Minus Vomitus ^3^	Soluble ESP ^4^	15K EVs ^1,5^	120K EVs ^4,5^
		Unique Peptides											
		R1	R2	R3	R4								
**Structural proteins**													
*Motor/cytoskeletal*													
Myosin heavy chain *	BN1106_s323B000258	17	17	23	24	●							
Paramyosin *	BN1106_s1922B000122	9	10	9	10	●							
Filamin	BN1106_s296B000186	4	3	4	2	●	●				●		
Filamin-C *	BN1106_s1515B000336	3	3	2	2	●						●	
Myosin heavy chain	BN1106_s323B000257	5	3	2	3	●	●						
Myosin heavy chain	BN1106_s3182B000117	8	8	0	3	●	●					●	
Alpha-actinin sarcomeric	BN1106_s4069B000247	8	10	3	10	●	●	●				●	●
Tropomyosin-2	BN1106_s4130B000080	1	2	1	5	●	●					●	
Actin	BN1106_s2907B000133	4	3	1	3		●	●		●		●	●
Titin	BN1106_s1119B000202	3	2	3	1			●					
Myosin regulatory light chain	BN1106_s527B000393	2	0	2	1		●						
Dynein heavy chain *	BN1106_s1314B000437	2	2	0	0							●	
Dynein light chain	BN1106_s949B000142	2	2	2	2		●	●				●	
Dynein light chain	BN1106_s1582B000145	1	2	2	1		●	●				●	
Dynein light chain	BN1106_s3147B000076	2	3	2	1		●	●				●	
*Cytoskeletal-binding/modifying*													
Spectrin beta chain, brain 4	BN1106_s4255B000066	3	5	2	1	●	●						
Calpain (C02 family)	BN1106_s204B000249	8	8	3	2	●		●				●	
Calponin homolog	BN1106_s2140B000163	4	4	1	1	●	●						
Gelsolin	BN1106_s2349B000191	1	1	3	6		●	●				●	●
Lymphocyte cytosolic protein 1	BN1106_s1403B000129	11	8	8	8		●	●	●	●		●	●
Gelsolin	BN1106_s2349B000188	5	5	3	8		●	●				●	●
Myophilin	BN1106_s3026B000096	2	2	0	0		●	●				●	
Adenylyl cyclase-associated protein	BN1106_s4290B000110	2	2	0	0		●					●	
*Extracellular matrix/cell adhesion*													
Heparan sulphate proteoglycan	BN1106_s25B000189	4	3	8	3	●	●				●		
Alpha 1 type IIA collagen *	BN1106_s849B000266	4	4	4	0	●							
Collagen alpha-1(V) chain *	BN1106_s457B000392	3	3	1	2	●							
Collagen alpha-1(V) chain *	BN1106_s2714B000202	4	3	3	1	●							
Fasciclin I-like protein *	BN1106_s1922B000120	6	5	4	2	●					●		
Collagen alpha-2(I) chain *	BN1106_s104B000457	4	3	2	2	●							
Collagen alpha-1(V) chain *	BN1106_s849B000265	2	3	3	2	●							
Collagen alpha-2(V) chain *	BN1106_s1528B000125	3	3	3	1								
Collagen type XV alpha *	BN1106_s26B000447	2	1	2	2								
**Signal transduction**													
*Signalling*													
Tyrosine 3-monooxygenase	BN1106_s3172B000053	2	2	2	1	●	●	●				●	
14-3-3 epsilon	BN1106_s686B000273	2	3	1	0	●	●	●		●		●	
cAMP-dependent protein kinase *	BN1106_s2316B000078	2	2	0	3							●	
Bcl-2 homologous antagonist	BN1106_s5167B000050	2	2	0	1		●						
*Calcium-binding*													
CaBP4	BN1106_s214B000744	3	5	4	7	●	●	●		●		●	●
Tegumental calcium-binding EF-hand protein	BN1106_s214B000742	2	2	3	2	●	●	●		●		●	
Tegumental calcium-binding EF-hand protein	BN1106_s214B000741	2	2	0	1		●	●				●	
Calmodulin-like protein 2	BN1106_s2277B000048	2	3	2	5		●	●		●		●	
*Ubiquitination pathway*													
Ubiquitin-activating enzyme E1	BN1106_s5276B000036	2	2	1	2	●	●					●	

## Data Availability

The data presented in this study are available in Supplementary Tables S1 and S2.

## Supplementary Materials

Supporting information can be downloaded at https://www.mdpi.com/article/10.3390/ijms23105525/s1.
