# Supplementary material for: Fasciola hepatica Gastrodermal Cells Selectively Release Extracellular Vesicles via a Novel Atypical Secretory Mechanism
Source: Int J Mol Sci. 2022 May 15;23(10):5525. doi: 10.3390/ijms23105525 (PMC9143473; doi:10.3390/ijms23105525)
Supplement: Supplementary file 1 [file ijms-23-05525-s001.zip › ijms-1700346-supplementary.pdf]

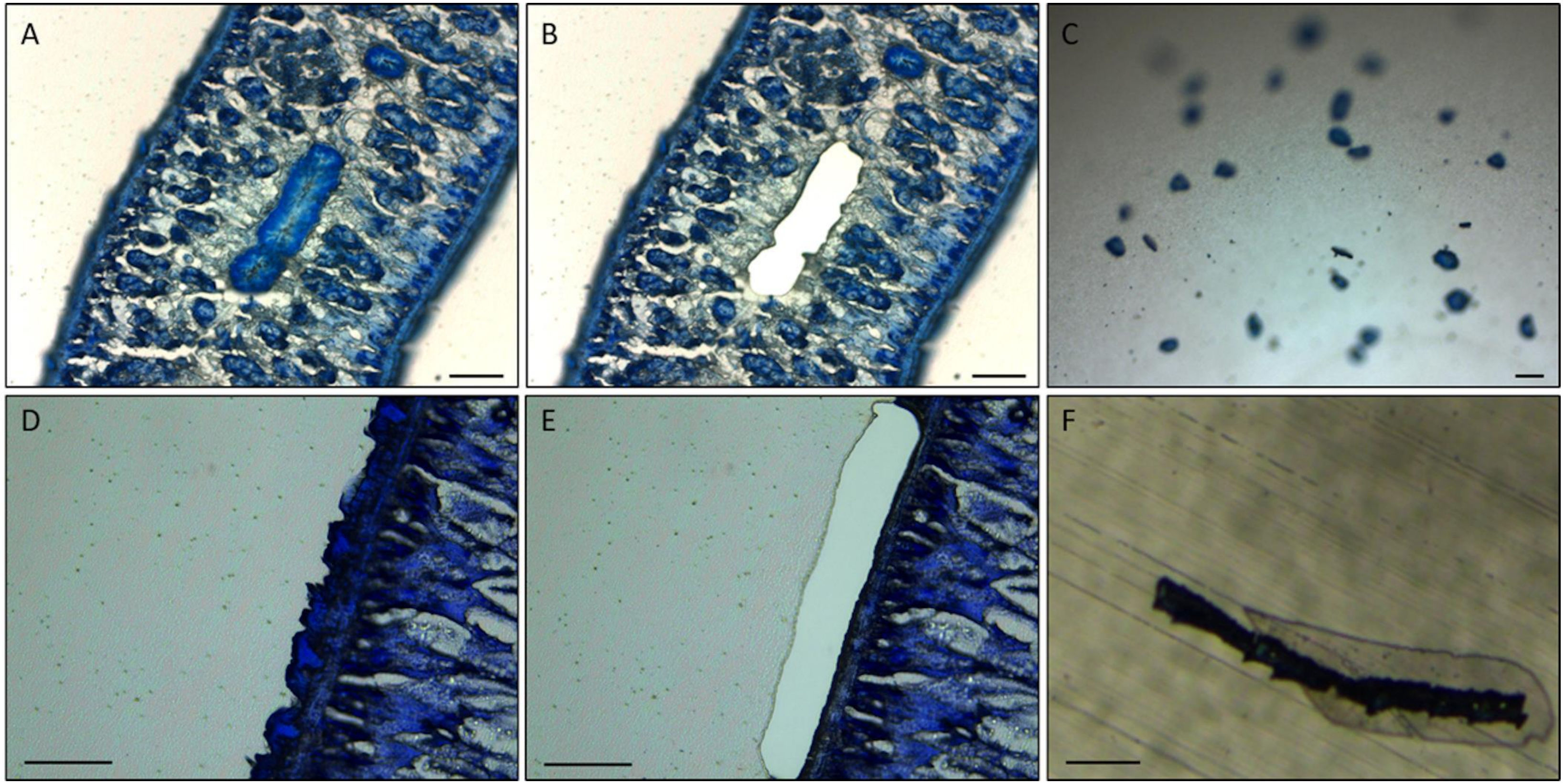


**Figure S1.** Sections of adult F. hepatica (stained with 1% toluidine blue) before and after laser microdissection. The left-hand panels show the gastrodermal cells (A) and tegument (D) before laser microdissection whilst the centre panels show the same sections with the gut (B) and tegument (E) removed. The right-hand panels show the recovered gastrodermal cells (C) and tegument (F). Scale bars, 100 μm.

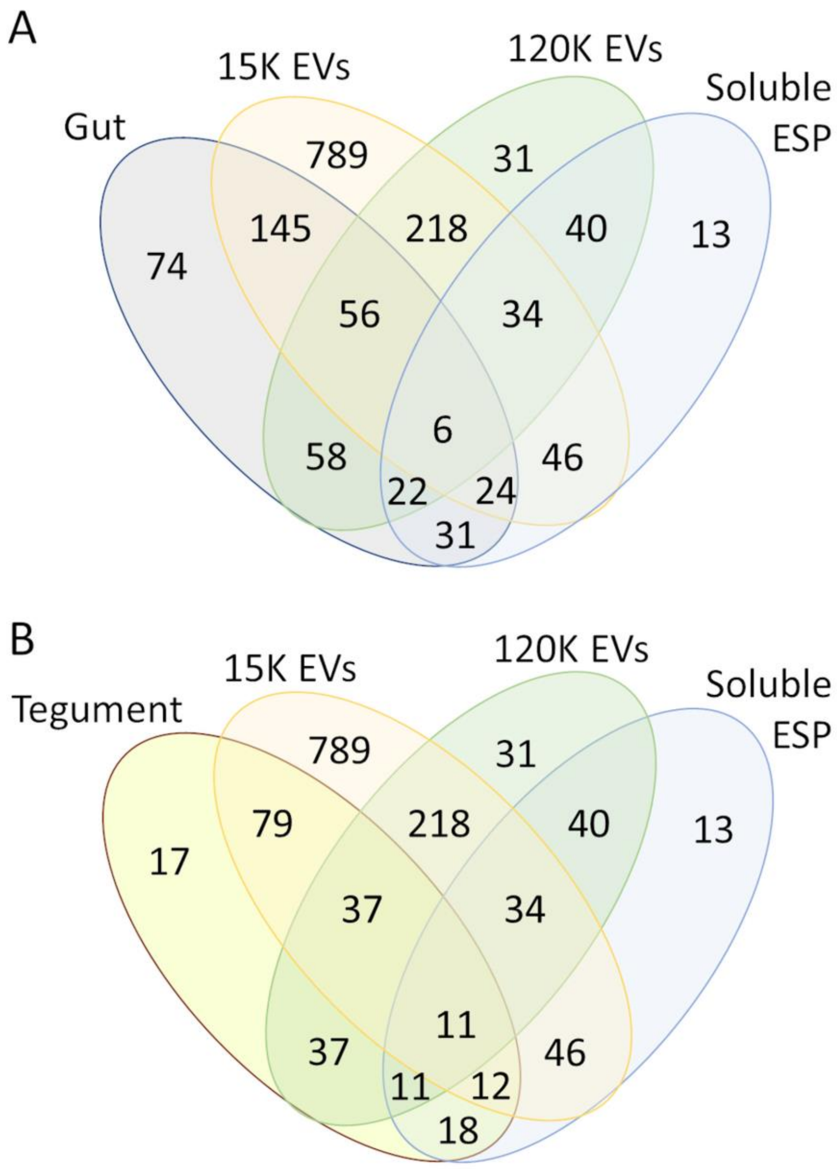


**Figure S2.** Venn diagrams showing the number of proteins shared between (A) the gastrodermal cells (gut), 15K extracellular vesicles (EVs), 120K EVs and soluble excretory/secretory proteins (ESP) and (B) the tegument, 15K EVs, 120K EVs and ESP. Gastrodermal cell, tegument and 15K proteomes are from this study. 120K EV and ESP proteomes are composed of data from Cwiklinski et al. [17] and de la Torre-Escudero et al. [24].
